# The TRANScending Love Arts-Based Workshop to Address Self-Acceptance and Intersectional Stigma Among Transgender Women of Color in Toronto, Canada: Findings from a Qualitative Implementation Science Study

**DOI:** 10.1089/trgh.2018.0040

**Published:** 2019-02-12

**Authors:** Carmen H. Logie, Ashley Lacombe-Duncan, Yasmeen Persad, Tatiana B. Ferguson, Dahlak Mary Yehdego, Shannon Ryan, Monica Forrester, Catherine Moses, Adrian Guta

**Affiliations:** ^1^ Factor-Inwentash Faculty of Social Work, University of Toronto, Toronto, Canada.; ^2^ School of Social Work, University of Michigan, Ann Arbor, Michigan.; ^3^ Women's College Research Institute, Women's College Hospital, Toronto, Canada.; ^4^ Black Coalition for AIDS Prevention (Black CAP), Toronto, Canada.; ^5^ Maggie's: Toronto Sex Workers Action Project, Toronto, Canada.; ^6^ Dalla Lana School of Public Health, University of Toronto, Toronto, Canada.; ^7^ School of Social Work, University of Windsor, Windsor, Canada.

**Keywords:** HIV, internalized stigma, intervention, prevention, transgender women

## Abstract

***Purpose:*** Transgender (trans) women of color's HIV vulnerabilities are shaped by social exclusion and intersectional stigma. There is a dearth of tailored HIV prevention interventions with trans women of color in Canada. The objective of the study was to explore trans women of color's HIV prevention priorities and to pilot test an intervention developed from these priorities.

***Methods:*** We conducted a qualitative implementation science study to develop HIV intervention strategies with trans women of color in Toronto, Canada. First, we conducted a focus group with trans women of color (*n*=8) to explore HIV prevention priorities. Second, we held a consultation with trans women of color community leaders (*n*=2). Findings informed the development of the TRANScending Love (T-Love) arts-based workshop that we pilot tested with three groups of trans women of color (*n*=18). Workshops were directly followed by focus groups to examine T-Love products and processes.

***Results:*** Focus group participants called for researchers to shift the focus away from trans women's bodies and HIV risks to address low self-acceptance produced by intersecting forms of stigma. The community leader consultation articulated the potential for strengths-focused arts-based approaches to address self-worth. T-Love participants described how workshops fostered self-acceptance and built connections between trans women of color.

***Conclusions:*** Findings demonstrate the feasibility and acceptability of an arts-based strategy with trans women of color to elicit group-based sharing of journeys to self-acceptance, fostering feelings of solidarity and connection. Providing opportunities for dialogue and reflection about individual and collective strengths may reduce internalized stigma among trans women of color.

## Introduction

Transgender (trans) women, who represent a diverse group of people labeled male sex at birth who identify as girls or women or on the transfeminine spectrum,^1^ experience elevated exposure to HIV,^2–5^ shaped by exclusion in social, economic, and health care systems.^6–11^ Trans women of color are disproportionately affected by HIV; meta-analytic findings from 29 studies in the United States reported a higher HIV seroprevalence among Black trans women (56.3%) than among White trans women (16.7%).^3^ In Canada, HIV vulnerability has also been reported among trans persons^12,13^ and among African, Caribbean, and Black (ACB) Canadians at a population level.^14^ In 2016, over one-fifth of new HIV diagnoses were among Black Canadians,^15^ despite comprising less than 5% of the Canadian population.^14^ Despite the overrepresentation of ACB persons and trans women in HIV diagnosis, there is little known about the HIV prevention priorities of ACB trans women in Canada or other trans women of color who may experience shared HIV vulnerabilities.^11^ A qualitative study with trans women living with HIV in Canada described a trajectory of marginalization, including socioeconomic exclusion, violence, and a lack of trans-specific HIV prevention and support, which constrained trans women's access to the HIV prevention cascade.^16^

Stigma, a driver of population health inequity, is widely documented among trans people^17^ and people of color.^18^ Stigma is multilevel and operates at *structural* levels through exclusionary institutional policies and practices, such as health care, employment, and housing; at *community* levels in felt-normative stigma through awareness of negative norms, attitudes, and values; at *interpersonal* levels through mistreatment and violence in interpersonal interactions; and at *intrapersonal* levels through internalized stigma, whereby people accept society's negative attitudes toward themselves and their communities.^19^ Each stigma dimension reduces trans women's health care access.^20^ Stigma is intersectional: social identities (e.g., gender and ethnoracial identities) are mutually constitutive, tied to systems of privilege and oppression.^21–26^ For trans women of color, racism, sexism, and trans stigma intersect to contribute to elevated HIV vulnerabilities.^11,26^

Although there is a scarcity of HIV interventions developed by and for ACB trans women in Canada, U.S.-based interventions have been developed to address HIV among trans women of color.^27^ Trans Sisters Informing Sisters about Topics on AIDS (T-SISTA)^28^ was adapted from the Centers for Disease Control and Prevention (CDC) evidence-based intervention Sisters Informing Sisters about Topics on AIDS (SISTA)^7^ for heterosexual cisgender African American women to address Black trans women's HIV prevention. T-SISTA includes group sessions addressing ethnic and gender pride; HIV, sexually transmitted infections (STIs), sex, and drugs; communication and negotiation; and safer sex practices and skills. There is limited data on the effectiveness of the T-SISTA intervention.^29^ Girlfriends is another intervention designed for trans women that addresses vulnerabilities and strengths at individual, interpersonal, and structural levels and has been evaluated with ethnoracially diverse trans women.^30^ Resilience and empowerment may contribute to health care engagement and positive health outcomes among trans women,^31,32^ and warrant further attention in HIV preventive strategies.

There remains a paucity of HIV interventions for ACB trans women in Canada.^7^ The applicability of interventions developed for African American trans women in the United States, such as T-SISTA, has not been explored with trans women of color in Canada—a context with different social and health care systems as well as historical and cultural experiences.^33^ Pilot testing and adapting interventions to ensure cultural and contextual relevance are a key facet of implementation science,^34,35^ and qualitative methods can provide important information on content and contexts that shape implementation.^36^ The article objectives are to describe the process of consulting with ACB trans women in Toronto regarding their HIV prevention priorities, and to discuss findings from pilot testing TRANScending Love (T-Love) with trans women of color in Toronto, Canada.

## Methods

### Study design and setting

This community-based research (CBR) study was conducted in collaboration with an ACB AIDS service organization, a lesbian, gay, bisexual, transgender, and queer (LGBTQ) community center, a sex worker agency, and ACB trans women community leaders in Toronto, Ontario, Canada's largest city.^37^ Qualitative methods are key to implementation science, providing the opportunity to examine factors important in enhancing or delivering health interventions.^36^ Consistent with CBR best practices for research with trans communities,^38^ engagement involved meaningfully working with ACB trans women community leaders in all research phases.^39–41^

Participants were purposively recruited by ACB trans women community leaders through their personal and employment-related e-mail networks, recruitment letters sent through e-mail and handed out in employment-related contexts, and through word-of-mouth between trans women, a form of snowball sampling. Community leaders utilized venue-based sampling, reaching out to trans women of color in their employment contexts. All trans women of color with whom community leaders had contact with during the recruitment phase were asked whether they wanted to participate. All trans women of color who expressed interest were invited to participate. Inclusion criteria included (1) identifying as a trans woman or having transfeminine experience, (2) identifying as African, Caribbean, Black, a person of color, or other racialized ethnicity, and (3) 18 years of age or older. All study participants completed a written informed consent process. The Research Ethics Board at the University of Toronto approved all study procedures.

### Multimethod approach to developing and pilot testing T-Love

#### Step 1: Focus group with ACB trans women

A focus group was held with ACB trans women (*n*=8) to explore HIV prevention and care priorities in October 2016. Focus groups are a recommended data collection approach in exploratory implementation science research.^36^ Participants were purposively sampled by ACB trans researchers, community agencies, and word-of-mouth. The focus group was cofacilitated by coauthors (CHL, DMY) with well-established ACB trans community connections, in collaboration with a trans community leader who was a research associate for this study (YP). This 90-minute focus group included a series of questions about the needs of ACB trans communities with respect to HIV prevention and the possibility of adapting T-SISTA, a behavioral intervention for HIV prevention among African American trans women.^42^ Questions included “If you were able to put together a small group of African, Caribbean, and Black transgender women in order to provide support, what kind of things would you offer” and “What information is important to include about sexual health, including HIV and other sexually transmissible infections?” After briefly describing T-SISTA, participants were asked questions such as “What are your thoughts about interest for T-SISTA among trans women here?”

#### Step 2: Consultation with ACB trans community leaders

After the qualitative analysis of the focus group transcript (described hereunder), the key findings were presented at a consultation with research team members (CHL, DMY, TBF, YP, SR), including two ACB trans community leaders in January 2017 to discuss next steps and conceptualize an intervention. Key informant interviews are another recommended strategy in qualitative implementation science work.^36^ Discussion questions included “In the focus group, we heard that people were tired of hearing about HIV, and we need to focus an intervention on self-love. What are your thoughts about this? What strategies might work?”

#### Step 3: Developing T-Love

Focus group and consultation findings resulted in the decision not to adapt T-SISTA due to its explicit HIV focus. The team instead drew from the qualitative findings to develop T-Love, a multimethod arts-based workshop focused on intrapersonal and social drivers of HIV vulnerability, including self-acceptance, self-esteem, strategies for coping, and social support. Arts-based research approaches provide the opportunity for participants to produce, interpret, and communicate about lived experiences through visual, written, and performance methods, producing knowledge and insights.^43,44^ These approaches allow participants to notice and communicate experiences in new ways, including those that are stigmatized and hard to explain.^45,46^ As such, arts-based approaches may be particularly relevant to identifying and addressing the complexity of stigma^47^ and empowering gender minority populations to enact intrapersonal, interpersonal, and social change.^48–50^

**FIG. 1.**
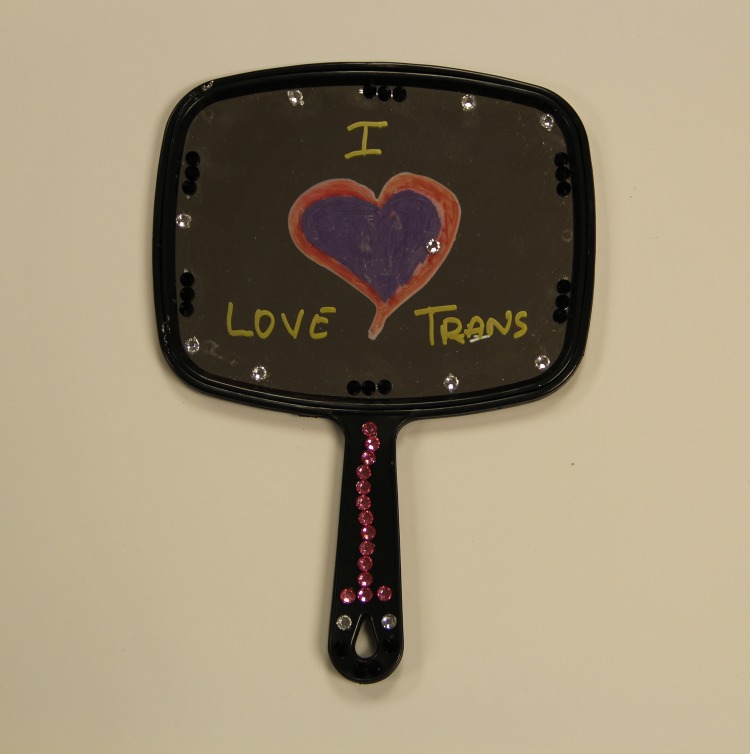
T-Love workshop mirror activity. T-Love, TRANScending Love.

**FIG. 2.**
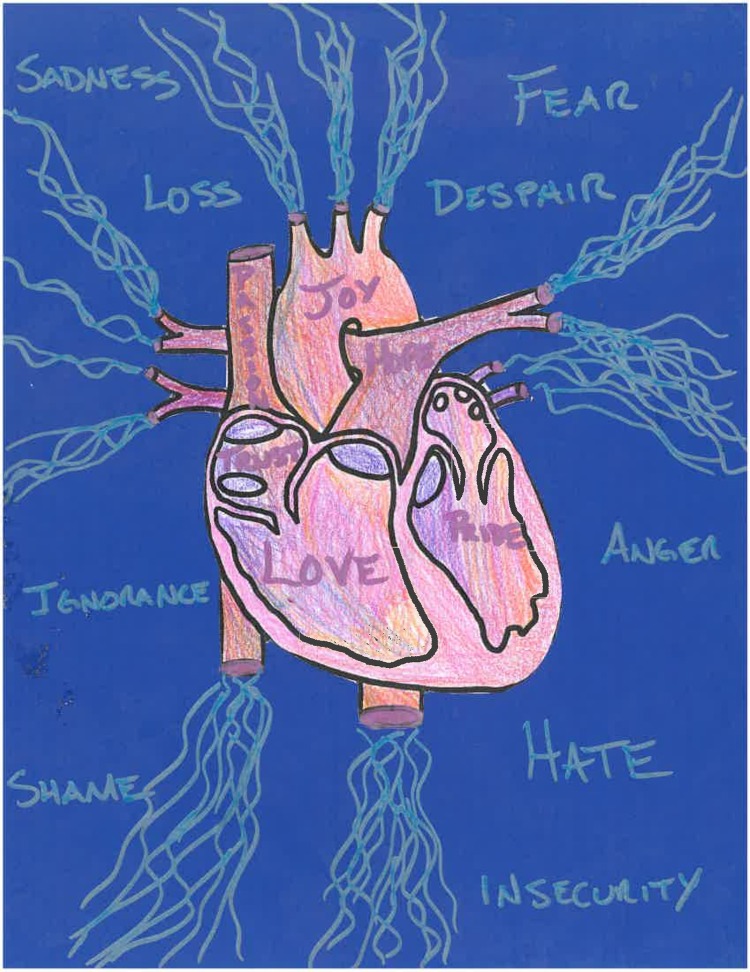
T-Love workshop anatomical heart activity.

T-Love comprised three arts-based approaches in a group setting: (1) hand-held mirrors with glass markers; participants were encouraged to reflect on their strengths and to write messages of self-love and affirmation (see Fig. 1 for example), (2) anatomical drawings of hearts to write and draw emotional blocks and coping strategies (example with Fig. 2), and (3) blank “you are loved” affirmation cards to write supportive messages to other trans women. The objectives of T-Love were twofold: (1) to encourage self-reflection on journeys to self-acceptance and stigma coping strategies and (2) to provide an opportunity for solidarity and connection between trans women of color.

#### Step 4: Pilot testing T-Love

We conducted three T-Love workshops in June 2017. Each workshop was ∼3 h; this included 60–90 min for the arts-based approaches, a 15–20 min break, followed by a 60–90 min focus group. One workshop was each held in (1) an ACB AIDS service organization, (2) an LGBTQ agency, and (3) a sex worker agency. Each workshop was preceded by completion of a brief demographic questionnaire, which collected data about participants' age, education, ethnicity, gender identity, social and legal gender affirmation, sexual orientation, immigration status, marital status, employment, HIV and STI testing history, and HIV status. Participants had the choice of which approach(es) they wanted to partake in, and also were informed they did not have to participate. Although originally intended to be conducted with ACB trans women, feedback from the community partnering agencies and ACB trans community leaders discussed that the workshop could be helpful for trans women of color generally, so three workshops were expanded to include Indigenous and trans women of color. One White participant came to one of the workshops; following the guidance of the trans team leaders we allowed her to participate.

Directly after the arts-based workshop, we conducted a focus group where participants were invited to share and describe the art they created, followed by semistructured open-ended questions about their experience of the workshop, and recommendations for further workshop development and HIV prevention strategies for trans women of color. All participants were asked whether they wanted to display and describe their art pieces (heart and mirror), could read their affirmation aloud, and were then asked to share their experiences creating the art, and recommendations for HIV prevention programs for trans women of color. Specific prompts and questions included “Please describe what you wrote on your mirror, and, or/and describe your heart?” “What was this experience like for you?” and “What recommendations do you have for this or another program to better meet your needs?” Each focus group and workshop participant received an honorarium of $50 per person. The focus group, consultation, and arts-based workshop focus groups were audio-recorded and transcribed verbatim.

### Data analysis

Participant demographics were summarized using proportions for categorical variables (e.g., ethnicity) and mean and standard deviation for continuous variables (e.g., age). Qualitative data were analyzed using narrative thematic analysis,^51^ facilitated by the use of NVivo 11 (QSR International Pty Ltd., 2012). Narrative thematic analysis involved one coder (CM) developing initial first-level codes, closely descriptive of the transcripts, including codes directly referencing the language of participants (*in vivo coding*).^52^ The code list was then applied by and refined iteratively by three other team members (ALD, AG, CHL) to increase the rigor of the analysis. Trans community leaders (*n*=3), including research associates for T-Love and article coauthors, provided debriefing and interpretation of findings, a type of member checking involving data interpretation by people with a shared lived experience to the participants.^53^

## Results

### Participant characteristics

A total of 28 trans women participated in the development (*n*=10) and pilot testing (*n*=18) of T-Love. The preliminary focus group (*n*=8) was followed by a consultation with ACB trans women community leaders (*n*=2) who had extensive experience working in social service provision with trans women. T-Love workshop participants (*n*=18) included a diversity of ethnoracial backgrounds, including Caribbean (*n*=6, 35.3%), Latinx (*n*=3, 18%), and Indigenous (*n*=3, 18%) persons. Approximately two-thirds of T-Love workshop participants identified their gender identity as woman (*n*=12, 66.7%), with five participants (16.7%) identifying their gender identity as other or *Two-spirit,* an identity to describe third or fourth gender people in Indigenous cultures (e.g., First Nations, Métis, Inuit, American Indian, and Alaskan Native) across North America.^54^ It can be understood as both gender identity and sexual orientation, thus was asked in relation to both facets of identity (Table 1).

**Table 1.** Sociodemographic Characteristics of TRANScending Love Workshop Participants (*n*=18)

Variable	Participants (*n*=18), *n* (%)
Age mean (SD)	32.36 (9.40)
Education (*n*=18)	
Less than high school, some college	6 (33.3)
College, some university	6 (33.3)
University	6 (33.3)
Ethnicity (*n*=17)^a^	
African	2 (11.8)
Caribbean	6 (35.3)
Latinx	3 (17.6)
Asian	1 (5.9)
South Asian	1 (5.9)
Indigenous	3 (17.6)
White	1 (5.9)
Gender identity (*n*=17)^a^	
Woman	12 (66.7)
Two-spirit other gender diversity^b^	5 (27.8)
Daily lived gender (*n*=18)	
Woman	15 (83.3)
Sometimes woman, sometimes man, non-binary or other gender	3 (16.7)
ID matches gender identity	
Yes	6 (35.3)
No	9 (52.9)
I have no documents	2 (11.8)
Sexual orientation (*n*=18)^a^	
Heterosexual	7 (38.9)
Bisexual	15 (19.0)
Queer	5 (6.3)
Two-spirited	2 (11.1)
Openminded	2 (11.1)
Immigrant (*n*=18)	
Yes	12 (66.7)
No	6 (33.3)
Marital status (*n*=18)	
Married/living together	5 (27.8)
Dating, not cohabiting	8 (44.4)
No current partner	5 (27.8)
Employment (*n*=18)	
Full time	2 (11.1)
Part time	2 (11.1)
Not employed	3 (16.7)
Self-employed	3 (16.7)
Student	2 (11.1)
Receiving social assistance	6 (33.3)
Ever tested for HIV (*n*=17)	
Yes	15 (88.2)
No	2 (11.8)
Ever tested for STI (*n*=17)	
Yes	12 (70.6)
No	5 (29.4)
Living with HIV (*n*=18)	
Yes	3 (16.7)
No	14 (77.8)
Prefer not to say	1 (5.6)

Percentages represented in the table are calculated based on reported values.

^a^ Participants were able to report more than one response with respect to ethnicity, gender identity, and sexual orientation. However, each participant only chose one response, thus, the categories are mutually exclusive.

^b^ Two-spirit is a North American Indigenous term that is understood as both a gender identity and sexual orientation, thus was asked in relation to both facets of identity. Some participants chose the response option “other” with respect to their gender identity.

STI, sexually transmissible infection.

### Focus group findings

Focus group participants critiqued how researchers and society at large were focused on trans women's sexuality and HIV risk (Table 2). As a focus group participant commented, “Anything about trans is always about sex…when do we stop becoming somebody's play toy?” Participant's connected this narrative to broader sexual scripts prevalent in media: “We're so overly sexualized on media, on TV, and through everything that we're like not human… That's how they [my partners] look at me. I'm just a sexual object.”

**Table 2.** Participant Quotes Describing Intervention Priorities for Trans Women of Color in Toronto, Canada (*n*=28)

Overarching theme	Focus group (*n*=8)	Community leaders (*n*=2)	T-Love workshop participants (*n*=18)
Need to focus on self-acceptance and the whole person beyond HIV (intrapersonal dynamics)	My concern would be mind, body and soul, overall wellness of individuals and facilitating growth of the person. HIV and everything else is there, yeah, and it's important…But what about the person? We say safe sex, but safe sex is not walking up to a stranger and just, oh, “do you have a condom?” It's not like that. In these situations, how do you value yourself? If we don't have people standing behind us to say that we can be loved, we're dealing with high rates of HIV and STIs in our communities. One time I was yearning so much to be loved that I thought sex was love.	With this activity, first we have to look about self-acceptance, self-love. After they've created their beautiful heart map and they feel empowered, they feel like they've presented such a masterpiece, then we can really touch on about their general health. So, the mapping was really about mental health and emotional stability, and they're now positioned to tackle something as tough as this (HIV). People are going to need time to really think about brainstorming where the journey is starting, and then reflect on whatever they're going through and how they're addressing, how they're coping. And then start to put things, like oh, we may have art supplies, magazines, whatever, glitter, start to really decorate your map.	I think that's why these exercises were so valuable, is that these are connected to our self-worth, the things we accept in our lives. If we don't think of ourselves of any worth or value, we'll let people hurt us, we'll let people have sex with us without condoms, or we'll do things that are risky for our health. So everything is interconnected. Some of the self-love topics that we touched on today we only went into the surface of them. I think it's something that we, as trans women, need to tap into more.
Diversity of trans women's experiences (interpersonal dynamics)	When we look at privilege in our community, it's so different. When I worked the streets and I did drugs and I needed a fix, I think I've opted to have no condoms. I know people do things because of situations. We have to look at the fact that some people may already come into this group with HIV. Some people may be sex workers. Some people may be older. Some persons might be new transitioning and not received as the ideal trans person.	How do Black people identify within the trans community? Race is a form of trauma for a lot of us who are barely moving forward. Embracing your identity. Really emphasize the essence of being trans. What does that mean for you, being trans and then also being Black? You have trans with an asterisk, that means various ways people can identify as trans.	There's not a lot of space where trans and nonbinary people can meet, and be free, and the spaces are very limited. And, there's certain things that we can't talk about and there's certain things that we can, and I feel like we need to start creating spaces around like language, we need to start creating spaces around body image, and self-worth, what's surgery looks like, what it doesn't look like, or, self-motivation. It's not only representation on trans people, but taking into account the intersection of realities of trans people. There are trans people of color. Trans people who are nonbinary for example. Are they represented within mainstream representations?
Strengths-based and empowering approach (community)	I think there needs to be more campaigns around trans people's bodies and that they're beautiful, and empowering. Because when we look at media and we look at television, it's always bashing trans women, and most often trans women of color. So, where are we getting our empowerment from? We all deserve a balanced, joyful life. We have to learn how to love ourselves first. And modules have to be put into place to teach us how to love ourselves first, so that we can get that balanced life, because it's not going to be from somebody else. Stop stigmatizing trans women of color on the lifestyles they live, the work they do, the survival they have to do. We need to embrace that and validate each and every one of our lives, we are important. Are we doing that outreach among each other and empowering each other at the same time?	Don't underestimate the people who are coming. Sometimes these people haven't been to a group, or they have. And sometimes they know more than I do, and they could teach me a lot of stuff. So, don't underestimate the woman. A lot of work that I do it's not HIV specific, but it's around anti-oppression, anti-racism, and going to practitioners and going to colleges, and I'm talking about diversity and I'm talking about inclusion. Someone else's journey, it's going to be inspiring. We're not only empowering them, but we're now giving them platform so other people see their work.	I don't think any of the available programs are aggressive enough to challenge the issues that the trans communities are facing in terms of, for example, programs that are catered specifically to empowerment. That everyone's voices are heard and respected, and everyone feels empowered in that space in some way, and leaves feelings some sort of empowerment, is really important. Doing more exercises where we really help each other, lift each other up, that's really important. Being able to rise each other up more, bring each others' spirits up and bringing people's self-worth up. It's really nice to share stories from people just like me, cause sometimes it's hard to find people that's actually been through the same thing and understand what you're going through, so you don't feel alone. What was important for me is the kind of bond with this kind of sisterhood that was established in this group.
Trans specific information, awareness and visibility (community)	There needs to be more awareness of trans' bodies. Working with women in the last couple of years and a lot of my peers that died over the last 30 years, a lot of it was because there was nothing to educate trans people on their bodies. Some feel forced into sex work. Some trans women do embrace sex work. It's having education around how not only to protect yourself from STIs and HIV, but how to ensure that it's not affecting your mental health or you're not increasing drug use in order to make a couple of extra dollars. These things intersect. It should be up-to-date in terms HIV, PrEP and PEP info sessions on that.	What I actually hate about doing sexual health work is that they use stats and figures and examples from things that do not relate to all trans women, so they're trying to generalize, use their findings in the research, generalize the experience. You need to sort of find out what that person's position is, and then see how compatible it is to the research. I definitely think the community needs more HIV resources, so that's what appeals to me most, having this information illustrated and available to other folks, so that they can explain it.	The more we can create topics or themes around sexual health. And we have so many wonderful sex workers in our community that have so much capacity to talk about those stories, as long as we have a host that's a sex worker we can always bring it back to sexual health. Just having those conversations, bringing it out of the shadows, and talking about it as normal, and there's nothing wrong with whatever you choose, as long as you're doing it safely, and look after yourself and others. We need trans representation in all communities. Every organization in this city we need to have a trans representation- hospitals, community center, government, police.
Address survival needs (structural)	Offer employment supports. A lot of trans women leave home very early, they lack education, so employment support would be connecting them with training opportunities, develop skills, also job placement. Having housing workers to help with people who are just starting to transition to helping them feel more comfortable. Employment discrimination: a lot of trans people are involved in sex work because they don't have any other place to get jobs, and we need to live, we have bills to pay.	So, once they've created these arts masterpieces, which is obviously very strong, and now we can touch on for persons who are HIV positive, what does that mean for them? How are they surviving? How regularly are they taking their treatments? The complexities of people trying to survive and access any kind of [HIV] care is crazy. There's also the survival, trying to actually live. Transphobia impacts people, and imagine if you're denied [health care]. That's such a critical state.	For me, it wouldn't be specifically titled “sexual health”, which I wouldn't want to come to. I want to come to something that would bring about change in my life. For example, I would want to come to a event of employers willing to hire trans people. There's a lot of trans people up there, who's very smart people but they hook up in drugs and prostitution because they said “the only way we can survive”, because if you dress nice, and go for a janitorial job, any work, so only to see maybe you look trans, maybe your ID don't match you, who you are, so I don't get accepted because the only simple point “I'm a trans woman”.
Address discrimination and gender-affirmative practice in HIV information and care programs (structural)	In terms of transitioning, especially to trans women, I would be sending them to very trans-friendly doctors who they can openly discuss everything with, not just throughout the process of starting hormones, but the impacts on your body, the impacts on your love life, the emotional component.	There's a lot of background work before you're going to develop a group like this, where women can be freely triggered. I'm very direct. So, the girls that I normally talk with don't have a problem in saying they're not coming back here.	I was invited to speak about trans women and HIV. And when I got there they start talking about studies about cisgender women and men, and gay people, and I was so mad. I have to speak out for my community, and I said to the person, “I feel discriminated, because you invited me here to talk about the trans community when you don't have nothing about trans community here.”
	Something that I felt when I was working in a culturally specific community, I'm not going to name any agencies, but I felt like I had to educate people. That is not my job to do though. And we need to come from the anti-oppression thing when we're working with communities.	Really equip staff with knowledge around trans sensitivity and trans inclusion. How do you create a safe dynamic when interacting with the front desk? It's really about creating safety. Because, now you're in the community and you notice that. They'll misgender you or call you by a name that you don't identify with.	Some organizations need to stop misleading the trans community. By using terms like “trans-inclusive”, but when you go there's nothing there for you. I work with this one organization who said they were trans inclusive, and they didn't know what transgender was! They thought a trans woman was a drag queen. And I didn't know if to laugh or if to be angry.

PrEP, pre-exposure prophylaxis; PEP, post-exposure prophylaxis; T-Love, TRANScending Love.

Focus group participants encouraged the research team to consider other parts of trans women's lives, beyond sexual health: “What about my life? What about me? What about what I want to be?” Another suggested, “Body positivity…and that's something that I feel has not been embraced as yet by program coordinators.”

Moreover, focus group participants articulated the need for empowering approaches to foster self-acceptance to advance trans women's health:

There's not enough outreach in communities to really empower trans women of color to love their bodies and to be safe. When you don't have that empowerment and you don't have those resources or outreach, of course, women are at higher risk of HIV and STIs.

The issue of needing positive affirmation was raised not only in relation to trans identity, but also in relation to ethnoracial identity, “I think we need to do a lot more education and empowerment within the communities that we are valid as human beings. We are valid as who we are as people of color.” As detailed in Table 2, participants also called for interventions to address survival needs such as housing and employment, to reduce stigma and promote gender-affirming care, and to foster empowerment among trans women of color.

### Intervention development

After describing the focus group findings already detailed, trans community leaders (*n*=2) articulated: the importance of focusing on self-love; the need to address intersectional identities of being both a person of color and trans, among other experiences; concerns regarding body mapping; the importance of building on the strengths of trans women of color communities; and the need to share arts-based products with the community (Table 2). Trans community leaders confirmed focus group participant's suggestions to focus on self-acceptance, “I feel like a lot of people, particularly Black people, they're a bit ashamed of their transness until you reach a certain point. So, talk about self-love, where do you start your journey, and what is transitioning for you?” Participants felt there could be benefits of sharing these journeys to self-acceptance in a group format. Consultation participants also discussed the need to consider the effects of stigma and discrimination at the nexus of intersecting identities, “How do Black people identify within the trans community? Part of it too is going to talk how race is a form of trauma for a lot of us who are barely moving forward.”

Body mapping, an approach where bodies are physically traced onto large pieces of paper, was suggested to consultation participants as a potential arts-based intervention as it has been used in HIV research^55^ and with sexual and gender minority populations.^56^ Consultation participants were concerned this approach could be triggering due to its focus on the physical body, “The word ‘body’, I'm just thinking, for trans people, who feel all kinds of discomfort, it's maybe a word to think about replacing. Instead of saying body mapping, what do you think? It could be triggering for some people.” The other participant corroborated this feeling, “Body, as soon as people hear the word body, in a trans context, they are triggered.” Although participants decided against body mapping, they agreed arts-based approaches would facilitate self-expression.

Community leaders also suggested the best length of an intervention (3 h), number of sessions (one), and timing (evening). The PI reflected on this information and proposed and brainstormed the three T-Love activities to the community leaders, which were codeveloped. The use of the anatomical heart, representing self-love, serves as an “empowerment map” in guiding understandings about emotions and coping. The mirror exercise was developed as a way to support women's love and compassion toward themselves. Finally, the affirmation cards were chosen to honor trans women's strengths and provide mutual support.

### Workshop feedback

Workshop participants described how the arts-based activities provided the opportunity for reflection on journeys to self-acceptance, in addition to the chance to build connections and experience mutual support with other trans women of color (Table 2). Participants expressed that the mirror exercise allowed persons to think about themselves in a different and less superficial way, “This is a really good exercise that makes me feel how happy I am, how I am proud about myself. This exercise makes me think about myself more than what I'm seeing in the mirror.” This facilitated gaining new perspectives, that, in turn, provided hope, “When you come into a space like this, and you talk about your own personal experience, you think ‘Ok, I never looked at it in this perspective’. There's a brighter, more fulfilling feeling of knowing a better way.” Others described feelings of self-acceptance after the workshop, “This whole group allowed me to actually think about who I am today and how far I have come, and my own self-love, I totally love myself more today than I ever have.”

Second, the group environment created a space of solidarity and sisterhood described as very supportive, “It is breathtaking to know that you can find trans women who would before knowing anything else about you, be open to share their stories, and be open to give you support.” Connecting with others who shared similar identities and life experiences was perceived as reducing isolation:

When I walk into a room and I see trans women of color, I always feel like, you get me, like no matter what, like we come into the world in a very unique way, and we can always relate to each other and I feel whatever you're feeling.

Finally, this type of workshop was discussed as providing a space to counteract negative messages from society:

I think the more we can celebrate our love for each other, and doing that work of self-care of saying we're beautiful to each other. That's something that I think is hard for us to do, ‘cause we get the opposite messages all the time. This was really magical.

Participant suggestions for future interventions (Table 2) include more opportunities to focus on self-acceptance, acknowledging the diversity of trans women's lived experiences, addressing survival needs with an emphasis on employment, and ensuring nondiscriminatory and gender-affirming health care and social spaces:

I really love this idea of continuing these sessions if you will, but building upon the capacity because it seems like people have a lot of experiences, a lot of education and I love hearing everyone speak.

To this end, although not a specific topic raised during the arts-based interventions, participants did discuss their sexual health, including HIV vulnerabilities, from a lens of trans exclusion:

If someone is not employed, obviously they would want to escort for example, to supplement their income. And what is the sort of, direct result of that action. Obviously ending up putting themselves at risk because of that action, right? So, let's not only focus on the end of result of the problem, as a result of multiple issues. Let's try to address these multiple issues.

## Discussion

In this qualitative study, we found that providing the opportunity to build self-acceptance and mutual support was an important first step in listening to trans women's voices. We also demonstrate the feasibility and acceptability of multimethod arts-based strategies with trans women of color to elicit group-based sharing of narratives of journeys to self-acceptance, in turn, fostering feelings of solidarity and connection. This development and pilot testing of T-Love, an innovative multimethods arts-based intervention in an urban Canadian context, suggests the promise of arts-based methods to foster dialogue and reflection about individual and collective strengths of trans women of color, in turn, potentially reducing internalized stigma. Participants identified lack of self-acceptance as a determinant of HIV vulnerability.

Key findings in this study included (1) interventions to address HIV should situate trans women's vulnerabilities in larger social and structural contexts of stigma and socioeconomic exclusion; (2) preferred intervention approaches are multilevel and can address *intrapersonal* (self-acceptance), *interpersonal* (relationships with others), and *community* (solidarity and empowerment; visibility of trans’ issues), and *structural* (survival needs, nondiscriminatory, and affirming services) social ecological dimensions.^57^ Findings can inform in-depth sustained approaches to address the complex pathways that exacerbate trans women's HIV vulnerabilities.^58^

Congruent with participant discussions of the hyperfocus of researchers and care providers on trans women's HIV risk, critical feminist scholars have similarly documented and critiqued the hypervisibility of trans women's HIV risk.^59,60^ Bauer and Hammond^60^ advocate for trans women's sexual health research to explore a deeper understanding of trans women's relationships with their bodies and for psychosocial support to include a focus on body image and self-esteem issues as impacted by systemic trans stigma. Although not an explicitly stated purpose of the workshop, some participants did discuss the topic of sexual health within social and structural contexts of marginalization. Participants advocated for interventions that address upstream determinants of HIV vulnerability, including increasing self-esteem/self-worth, but also employment and health care access.

Participants also articulated that HIV, survival, and well-being needs were interconnected. For example, participants noted a lack of safe affirming spaces and employment barriers. Many lacked legal identity documentation that reflected their gender identity, which may contribute to health care stigma^20^ and reduced self-esteem.^61^ Women's narratives reflect the concept of self-compassion, referring to mutual interactions between kindness, a sense of common humanity, and mindfulness.^62,63^ Strengths-based HIV prevention and care cascade interventions with trans women of color can challenge social exclusion and apply a holistic self-compassion approach to harness the strengths within trans women of color communities. However, approaches such as T-Love must be coupled with broader structural interventions to increase access to employment and gender-affirmative services for trans women.^19^

These interventions may not necessarily be framed from an HIV prevention lens; in fact, framing as stigma reduction and self-acceptance may broaden the focus from HIV to other social determinants of health. This approach maps onto calls for comprehensive HIV prevention initiatives for transgender persons that consider individual, interpersonal, social, and structural factors that shape HIV vulnerabilities.^8^ Community-based approaches that engage meaningfully with communities to identify their needs and proposed solutions can inform acceptable interventions grounded in diverse lived experiences.^40,41^ Our findings suggest that an arts-based intervention can lead to meaningful conversations about the interplay between stigma, self-worth, and HIV vulnerabilities among trans women of color. These conversations may shift awareness of sexual decision-making.

The results of this small pilot study should be interpreted in light of its limitations. The results of this study are not generalizable to the experiences of all trans women of color in Canada. Study participants live in a large diverse urban environment, unlike many trans people in Canada who live in rural environments.^64^ As such, they were likely more easily able to come together, may already be a part of trans and/or ACB communities, and were potentially more attuned to the issues their communities encounter. In addition, T-Love should be replicated with larger samples of ACB trans women, include more sessions (for instance, focused on other social determinants of health including employment and health care access), involve a longer follow-up (e.g., 6-weeks), and measure changes in self-esteem, stigma, and community connectedness. Osman et al.'s^65^ analysis of 62 arts-based health interventions documents a lack of clarity regarding outcome evaluations, suggesting that more rigorous T-Love interventions can also contribute to the knowledge base on arts-based health interventions.

Despite these limitations, findings suggest that T-Love was an acceptable intervention that facilitated self-reflection and community building. This unique study highlights the importance of CBR with ACB trans communities in Canada. Future studies could employ mixed methods and longitudinal approaches to T-Love, and expand to include HIV prevention sessions that integrate analyses of self-esteem and the social determinants of health.

Intersectional stigma based on trans and racialized identities contributes to low self-esteem and reduced access to social support: T-Love's innovative approach addresses these factors. T-Love was perceived as empowering and providing a space for solidarity between trans women of color. HIV vulnerabilities among trans women are also shaped by low self-esteem and social isolation, suggesting that T-Love's approach could be harnessed in HIV prevention strategies. Exploring issues that affect trans women of color in Canada through CBR can facilitate the development of tailored interventions to optimize health and well-being.

## Abbreviations Used

ACB: African, Caribbean, and Black
CBR: community-based research
LGBTQ: lesbian, gay, bisexual, transgender, and queer
SISTA: Sisters Informing Sisters about Topics on AIDS
STI: sexually transmissible infection
T-Love: TRANScending Love
T-SISTA: Trans Sisters Informing Sisters about Topics on AIDS
